# Integral Lipophilicity Studies with in Silico ADMET Parameter Analysis of Novel Pseudothiohydantoin Derivatives

**DOI:** 10.3390/ijms27146310

**Published:** 2026-07-15

**Authors:** Szymon Baumgart, Małgorzata Redka, Artur Słomka, Renata Studzińska

**Affiliations:** 1 Department of Organic Chemistry, Faculty of Pharmacy, Collegium Medicum in Bydgoszcz, Nicolaus Copenicus University in Toruń, 2 Jurasza Str., 85-089 Bydgoszcz, Poland; mredka@cm.umk.pl (M.R.); rstud@cm.umk.pl (R.S.); 2 Department of Hematology and Oncology, National Medical Institute of the Ministry of Interior and Administration, 137 Wołoska Str., 02-507 Warsaw, Poland; artur.slomka@cm.umk.pl

**Keywords:** lipophilicity, RP-HPLC, RP-TLC, pseudothiohydantoin derivatives, ADMET analysis

## Abstract

This article describes the assessment of the lipophilicity of 18 new pseudothiohydantoin (2-aminothiazol-4(5*H*)-one) derivatives using experimental and computational methods. Previous studies have shown that the tested derivatives are inhibitors of 11β-hydroxysteroid dehydrogenase type 1 and inhibit the metabolic activity of selected cancer cell lines. Lipophilicity, as log k_w_ and R_M0_, was determined using reversed-phase high-performance liquid chromatography (RP-HPLC) and reversed-phase thin-layer chromatography (RP-TLC), respectively. In both chromatographic methods, methanol served as a modifier of the mobile phase. The obtained R_M0_ values were higher than the log k_w_ for most of the compounds analyzed. The chromatographically determined lipophilicity parameters were compared with those obtained using the commonly used VCCLAB software: milogP, AlogPs, AClogP, XLOGP2, and XLOGP3. Among the analyzed calculation methods, the best agreement with experimentally determined lipophilicity (both log k_w_ and R_M0_) was demonstrated by milogP and AClogP values. All tested derivatives satisfied the Lipinski, Veber, Ghose, and Egan rules, indicating their favorable properties after oral administration. Additionally, in silico calculations of ADMET parameters revealed that most of the 18 compounds tested exhibited promising pharmacokinetic and toxicological properties, making them attractive drug candidates.

## 1. Introduction

Discovering new therapeutic agents, especially those used in treating cancer and metabolic syndrome, currently poses a significant challenge in medicinal chemistry. Both cancer and metabolic syndrome are presently the leading causes of morbidity and mortality worldwide [1]. Over the years, pseudothiohydantoin derivatives (2-aminothiazol-4(5*H*)-one) have been identified as a class of compounds that demonstrate potential in the treatment of these two conditions. Among them, compounds exhibiting cytotoxic activity against selected cell lines have been identified, including breast cancer (MDA-MB-231, MCF-7), colon cancer (HCT116, SW480, RKO), lung cancer (H460a), and osteomyeloma (SJSA1) [2,3,4,5]. Moreover, their pharmacological activity is also attributed to their interaction with 11β-hydroxysteroid dehydrogenase type 1 (11β-HSD1). This enzyme regulates cortisol levels in the human body and has been linked to the pathogenesis of metabolic syndrome and carcinogenesis [6,7]. Some derivatives from this group have been shown to act as selective 11β-HSD1 inhibitors; some have been clinically tested for treating obesity and type 2 diabetes, further emphasizing their therapeutic potential [8,9].

While discovering new treatment methods, in addition to synthesizing and identifying biologically active compounds, it is also important to assess physicochemical parameters, pharmacokinetic safety profile and toxicological characteristics.

Lipophilicity, expressed as the logarithm of the n-octanol/water partition coefficient (logP), is the most informative physicochemical parameter in discovering and designing new drug candidates. It provides essential information about the compound’s properties as a single descriptor. It directly influences every stage of the pharmacological pathway of the substance in the human body, from absorption, through distribution, metabolism, and elimination [10]. Lipophilicity influences, among others, permeability through biological membranes [11], drug potency [12], solubility [13], and binding to plasma proteins [14]. If a compound’s lipophilicity is too low, its water solubility increases, thereby reducing its ability to cross biological membranes [15]. In the case of permeability across the blood–brain barrier (BBB), small, lipophilic molecules readily penetrate the BBB, with higher lipophilicity leading to better permeation properties [16]. Conversely, higher lipophilicity favors the capture of molecules by efflux pumps and their expulsion to the other side of the membrane [17]. Furthermore, compounds with high lipophilicity are characterized by rapid metabolism and enhanced toxic [18,19]. Excessive lipophilicity is believed to reduce the selectivity of binding to the biological target, which promotes adverse effects [20]. Therefore, lipophilicity testing is an essential step in the rational design of bioactive compounds.

Determining the ADMET profile (A—absorption, D—distribution, M—metabolism, E—excretion, T—toxicity) is an essential step in the development of new drugs. The unfavorable pharmacokinetic profile and toxicity of new drug candidates (NDAs) are barriers that often contribute to failures in later phases of clinical trials and significantly increase research costs. According to reports from the late 1990s, almost half of NDAs were withdrawn for ADMET-related reasons [21]. This has led to intensive research development of ADMET parameter prediction and incorporating it as an integral part of the process to maximize optimization of screening tests [22].

This study aimed to evaluate the lipophilicity parameters of 18 new derivatives of 2-(cyclopentylamino)thiazol-4(5*H*)-one (**1**–**9**) and 2-(cyclohexylamino)thiazol-4(5*H*)-one (**10**–**18**) (Table 1) with proven 11β-HSD1 inhibitory and antiproliferative activity using chromatographic (RP-TLC and RP-HPLC) and computational methods. The compounds were also tested in silico for their physicochemical properties and ADMET parameters—parameters necessary to determine the substance’s pathway in the human body.

## 2. Results and Discussion

### 2.1. Lipophilicity Studies

Lipophilicity studies were performed for two series of pseudothiohydantoin derivatives: 2-(cyclopentylamino)thiazol-4(5*H*)-one (**1**–**9**) and 2-(cyclohexylamino)thiazol-4(5*H*)-one (**10**–**18**). The determination of this parameter was divided into two stages. Log k_w_ and R_M0_ values were determined in the first stage using RP-HPLC and RP-TLC chromatographic methods. Methanol was used as the organic modifier in both methods. Linear dependencies of log k_w_ and R_M0_ on methanol concentration were determined based on Equations (2) and (5). High values of correlation coefficients (r = 0.99 for RP-HPLC and r = 0.95–0.99 for RP-TLC) allowed the determination of the lipophilicity parameters log k_w_ and R_M0_ by extrapolation. The determined values are presented in Table 2.

**Figure 1:**
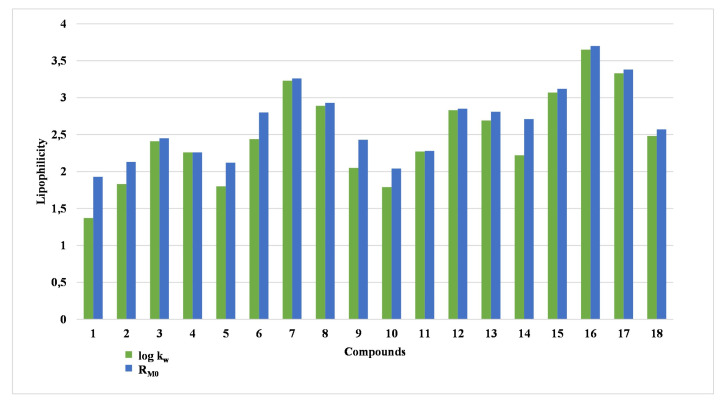
Correlation between log k_w_ and R_M0_ values for compounds **1**–**18**.

The analysis of the obtained results showed that the determined log k_w_ values, ranging from 1.37 to 3.65, are lower than the R_M0_ values, ranging from 1.93 to 3.70 (Figure 1). The exception is derivative **4**, for which identical values of log k_w_ and R_M0_ were obtained.

**Figure 2:**
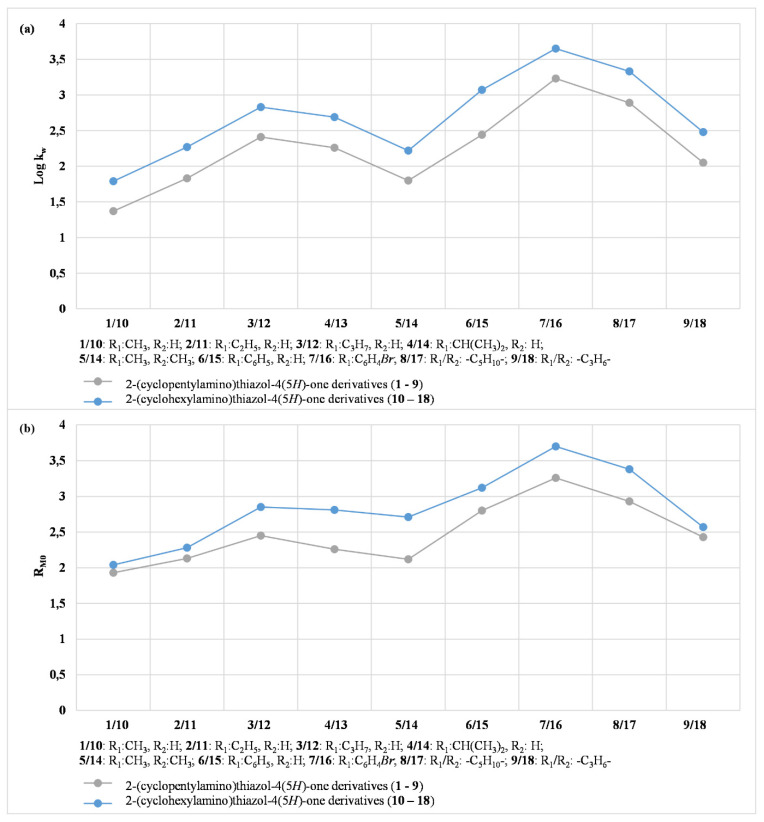
Relationship between (**a**) log k_w_ values, (**b**) R_M0_ values and substituents at the amino group and the C5 position of the thiazole ring.

Additionally, the results obtained using both methods revealed a relationship between the structure of the tested compounds and lipophilicity parameters. It was found that the type of substituent at the amino group and C5 of the thiazole ring influenced the log k_w_ and R_M0_ values. Higher lipophilicity values were obtained for the identical substituents at the C5 position for 2-(cyclohexylamino)thiazol-4(5*H*)-one derivatives than for 2-(cyclopentylamino)thiazol4(5*H*)-one (Figure 2).

Among the tested derivatives, the least lipophilic compound was 2-(cyclopentylamino)-5-methylthiazol-4(5*H*)-one (**1**; log k_w_ = 1.37; R_M0_ = 1.93), while the most lipophilic was 2-(cyclohexylamino)-5-(4-bromophenyl)thiazol-4(5*H*)-one (**16**; log k_w_ = 3.65; R_M0_ = 3.70).

For both series of 2-aminothiazol-4(5*H*)-one derivatives with unbranched aliphatic chains at C5 of the thiazole ring, it was observed that with increasing chain length: CH_3_ (**1**; **10**) < C_2_H_5_ (**2**; **11**) < C_3_H_7_ (**3**; **12**), the log k_w_ and R_M0_ values increase. However, in the group of compounds with branched substituents at C5, higher log k_w_ values were obtained for derivatives with an isopropyl substituent (**4**—2.26; **13**—2.69) compared with two methyl groups (**5**—1.8; **14**—2.22). A similar relationship was found for the value of the R_M0_ parameter.

In the case of derivatives with a phenyl substituent, an increase in lipophilicity was observed for 2-(cyclohexylamino)-5-phenylthiazol-4(5*H*)-one (**15**) compared to aliphatic derivatives. However, for 2-(cyclopentylamino)-5-phenylthiazol-4(5*H*)-one (**6**), a significant increase was observed only for the R_M0_ parameter, while the log k_w_ value was similar to that obtained for the derivative with a propyl substituent. Introduction of a bromine atom at the 4-position of the phenyl ring (derivatives **7** and **16**) resulted in a significant increase in the log k_w_ and R_M0_ values, making these derivatives the most lipophilic compounds among those tested.

Similarly to the previously studied derivatives [23], in the group of compounds containing a *spiro* system of thiazole and alicyclic rings, it was found that compounds with a less developed carbon skeleton (**9**; **18**) were less lipophilic than compounds with a more developed structure (**8**; **17**).

In the second stage of the study, theoretical lipophilicity parameters were determined: milogP, AlogPs, AClogP, XLOGP2, and XLOGP3, which were then compared with the values determined chromatographically (log k_w_ and R_M0_). The results are presented in Table 3.

Theoretical lipophilicity values obtained are characterized by similar relationships to those determined chromatographically. The lowest values for the tested derivatives were obtained in the XLOGP2 model, and the highest in the AlogPs model (except for derivatives **4**, **8**, **11**, **17**).

**Figure 3:**
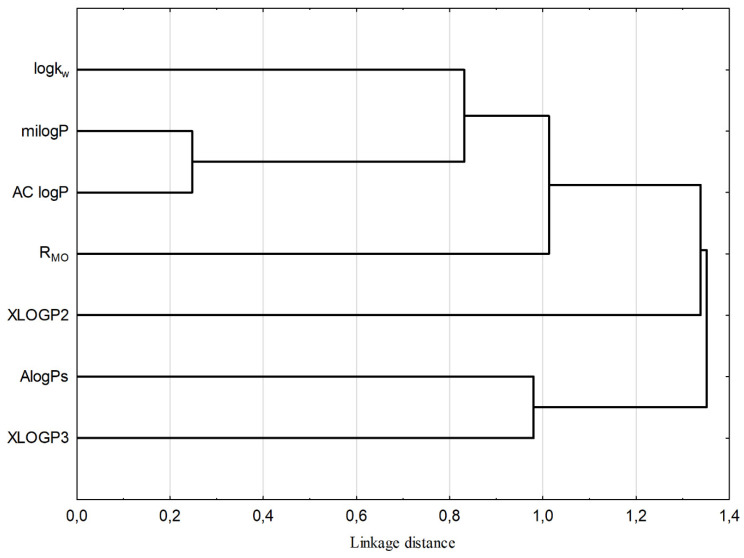
Dendrogram of similarity of lipophilicity values determined chromatographically and theoretically for the tested compounds.

Hierarchical cluster analysis (HCA) was used to compare experimental and theoretical values. As shown in Figure 3, the experimentally determined lipophilicity parameter values are most similar to the theoretical values of AClogP and milogP. These results imply that both of these parameters can be a reliable option for predicting lipophilicity for the tested pseudothiohydantoin derivatives.

In addition, Pearson and Spearman correlation matrices were created for lipophilicity parameters: log k_w_, R_M0_, miLogP, ALogPs, AClogP, XLOGP2 and XLOGP3 (Table 4). A scatter plot illustrating the relationships between two experimentally determined lipophilicity parameters (log k_w_ and R_M0_) is provided in the Supplementary Materials (Figure S1).

Most of the obtained results exceeded 0.9, indicating a very strong correlation between the outcome. The only exceptions are the correlations between R_M0_ and XLOGP3, and R_M0_ and AlogPs determined using the Spearman method, which fall within the range of 0.7–0.9, indicating a slightly lower monotonic correlation.

Due to the above results, conducting lipophilicity studies for pseudothiohydantoin derivatives turned out to be justified and necessary in the process of evaluating compounds as potential drug candidates.

### 2.2. Bioavailability—In Silico Prediction

Physicochemical parameters were calculated for 2-(cyclopentylamino)thiazol-4(5*H*)-one and 2-(cyclohexylamino)thiazol-4(5*H*)-one derivatives (**1**–**18**) in order to their oral bioavailability. The parameters necessary for the assessment are presented in Table 3 and Table 5 (milogP).

In assessing drug bioavailability following oral administration, the most widely recognized and frequently used criterion is the “rule of five” proposed by Lipinski. This rule assumes that a molecule readily available after oral administration should have a molecular weight below 500 Da, lipophilicity (ClogP) below 5, the number of hydrogen bond donors (HBD) ≤ 5, and the number of hydrogen bond acceptors (HBA) ≤ 10 [24]. Discovered that all tested derivatives did not violate any of the above-mentioned rules and thus met Lipinski’s rule of five.

In addition to Lipinski’s rule of five, other rules have been developed to assess drug-likeness. These include the Veber, Egan, and Ghose rules, which consider other physicochemical parameters influencing a compound’s oral availability. Veber’s rule requires that compounds meet two criteria to be considered potentially orally available. These parameters include a number of rotatable bonds of ≤10 and a polar surface area of ≤ 140 Å^2^ [25]. Egan’s rule also considers two criteria influencing bioavailability: PSA and AlogP [26]. Additional rule that helps assess a compound’s predisposition to oral bioavailability is the Ghose rule, which unlike the Veber and Egan rules, has a broader set of criteria the tested molecule must meet. A compound meeting all the criteria of this rule should fall within the following descriptor ranges: molecular weight (from 160 to 480), logP (from −0.4 to 5.6), molar refractive index (from 40 to 130), and number of atoms (from 20 to 70) [27]. All tested substances **1**–**18** also meet all the criteria of the Veber, Egan, and Ghose rules, indicating the potential for good oral bioavailability.

### 2.3. In Silico ADMET Prediction

#### 2.3.1. Absorption

In this study, the absorption of 18 tested compounds was assessed using parameters of aqueous solubility, Caco-2 (human colon adenoma cell) cellular permeability, absorption in the small intestine, and skin permeability. The obtained results are presented in Table 6.

The aqueous solubility of a compound is an significant parameter as it is used to optimize the composition of drug formulations subsequently [28]. The aqueous solubility of the tested derivatives was assessed using the LogS parameter (value expressed in mol/L). All 18 pseudothiohydantoin derivatives are characterized by poor aqueous solubility, with predicted LogS values ranging from −2.225 to −5.003. This means that when designing drug formulations, it will be necessary to use a cosolvent, salt formation, or prodrug to improve the solubility and availability of the compounds.

In the case of oral drug absorption, an important step is the assessment of permeability through the intestinal epithelium. For this purpose, the permeability parameter through a Caco-2 cell monolayer is determined. The Caco-2 cell monolayer shows structural and functional similarity to human epithelial cells and is widely used as an in vitro model to mimic intestinal absorption [29,30]. Caco-2 permeability in this study is expressed as the logarithm of the apparent permeability coefficient (logPapp). In the Caco-2 cell permeability model, compounds with a logPapp value greater than 0.9 are classified as having high permeability. According to the analysis performed, all synthesized derivatives can show high Caco-2 cellular permeability as the calculation results range from 1.459 to 1.619. Good Caco-2 permeability often correlates with significant intestinal absorption (HIA—human intestinal absorption) [31]. Intestinal absorption determines the percentage of a compound that is absorbed and is a comprehensive parameter, as it also considers metabolic processes occurring in the intestinal epithelium [32]. For the tested derivatives, the calculated HIA values are high, ranging from 90.929% to 94.414%. The lowest intestinal absorption values were obtained for derivatives containing a bromophenyl substituent at the 5-carbon position of the thiazole ring (derivatives **7** and **16**).

Determining the skin permeability parameter is important when transdermal drug administration is necessary [33]. The skin permeability coefficient was predicted as the skin permeability constant logKp. LogKp values greater than −2.5 suggest low penetration of the active substance through the skin. Of the tested derivatives, only compounds **9** (−2.432) and **18** (−2.436) showed logKp values greater than −2.5. For the remaining derivatives, the skin permeability values ranged from −2.503 to −3.077, indicating a low ability to penetrate the skin.

#### 2.3.2. Distribution

The distribution of the tested pseudothiohydantoin derivatives **1**–**18** was assessed using parameters such as the volume of distribution at steady state (VDss), unbound fraction (FU), blood–brain barrier (BBB) permeability, and permeability to the central nervous system (CNS). The obtained results are presented in Table 7.

Volume of distribution (VDss) is a key pharmacokinetic descriptor that, together with clearance, provides information on the drug’s administration schedule, as both parameters determine the average residence time of the drug in the body [34]. A low VDss indicates that the compounds remain more bound to plasma due to their good water solubility or high plasma protein binding. A high VDss, on the other hand, indicates a greater affinity of compounds to tissues, which is related, among other things, to high lipid solubility or strong binding to tissue proteins [35]. A VDss value is considered low when it is less than −0.15 log L/kg, while a value exceeding 0.45 log L/kg is considered high. The calculated VDss values of the tested compounds are in the range of 0.123 to 0.384, which suggests that the compounds are likely to maintain equilibrium, i.e., they will not accumulate excessively in tissues or circulate in the body in combination with plasma. The unbound fraction (FU) is also a crucial pharmacokinetic parameter, as it refers to the portion of the drug that is not bound to plasma proteins and, being free, can exert pharmacological effects by binding to its biological targets (enzymes, channels, proteins, or receptors) [36]. Furthermore, FU generally influences the pharmacokinetic profile, impacting factors such as glomerular filtration rate, total clearance, and hepatic metabolism [37]. The unbound fraction values for the 18 pseudothiohydantoin derivatives tested ranged from 0.19 to 0.618.

The blood–brain barrier (BBB) is a selective barrier that, on the one hand, allows the delivery of essential nutrients from the bloodstream to the brain, and on the other hand, protects it from the effects of potentially harmful substances [38]. Therefore, the ability to penetrate the blood–brain barrier and the central nervous system (CNS) is an important property in developing new drugs. For drugs developed to treat CNS-related disorders, crossing the blood–brain barrier is an essential and required feature [39]. In turn, for drugs whose pharmacological action does not involve the CNS, limited BBB penetration is desirable to avoid, among other things, side effects [39]. The results obtained for the tested compounds ranged from −0.208 to 0.242, suggesting that these compounds may have the potential to penetrate the blood–brain barrier. However, the results obtained are more variable for the more precise parameter of CNS permeability [39]. For derivatives **1**–**3**, **4**, **10**–**12**, and **14**, logPS (permeability surface area product) values of <−3 were obtained, which implies that they will not penetrate the central nervous system. The remaining tested derivatives are probably permeable to the central nervous system (logPS values > −3).

#### 2.3.3. Metabolism and Excretion

Analysis of the metabolism of the studied 2-aminothiazol-4(5*H*)-one derivatives included their potential interaction with cytochrome P450 enzymes, considering their ability to act as substrates or inhibitors. Acting as inhibitors suppress the action of a CYP450 enzyme, which can lead to restriction of the substrate (drug) metabolism, leading to increased concentrations and an increased risk of adverse events. Furthermore, information that a given compound is a substrate of CYP450 enzymes indicates that it may be metabolized by these enzymes. Enzymes of the cytochrome P450 family are responsible for the biotransformation of nearly 75% of currently used drugs [40]. The study determined the predicted interaction of the compounds with the most important CYP450 isoforms, namely CYP3A4, CYP1A2, CYP2C9, CYP2D6, and CYP2C19. Of these five enzymes, CYP3A4 plays the most crucial role in drug biotransformation, as it is responsible for the metabolism of nearly 50% of all drugs [41]. The results indicate that none of the tested compounds will be CYP3A4 inhibitors, while substances **6**, **7**, and **15**–**17** were identified as CYP3A4 substrates. Another important member of the CYP450 enzyme family is CYP2D6, as it is responsible for the metabolism of 25% of drugs and exhibits a high degree of polymorphism [42]. However, in the case of this enzyme, none of the analyzed compounds were classified as either inhibitors or substrates of CYP2D6. Another important enzyme from the CYP450 family is CYP1A2, which accounts for approximately 13% of the total content of these enzymes in the liver and plays a key role in the metabolism of drugs with a narrow therapeutic index, such as theophylline and clozapine [43,44]. CYP2C9 is another clinically significant enzyme, as it plays a role in the metabolism of 15% of commonly used drugs and is highly genetically polymorphic [45,46]. CYP2C19, in turn, is also characterized by a high degree of polymorphism, with nearly 49 allele variants identified, many of which affect the biotransformation of important drugs, including clopidogrel [47]. The results obtained for the last three enzymes described show that almost all derivatives do not exhibit inhibitory properties for CYP2 (excluding compounds **4** and **13**), CYP2C9 (excluding compounds **3** and **7**), or CYP2C19 (excluding compounds **6** and **7**).

The parameter used to assess excretion is total clearance, expressed as log (mL/min/kg). Total clearance consists of hepatic clearance, renal clearance, and clearance from other body tissues [48]. This parameter is key in determining doses and selecting dosing regimens [49]. The calculated total clearance for the tested derivatives ranged from −0.111 to 0.243. The obtained results for metabolism and elimination are presented in Table 8.

#### 2.3.4. Toxicity

The potential toxicity of the analyzed 2-aminothiazol-4(5*H*)-one derivatives was studied using parameters such as maximum tolerated dose, acute and chronic oral toxicity in rats, AMES toxicity, toxicity to *Tetrahymena pyriformis*, and minnow fish. The results for these parameters are summarized in Table 9.

The maximum recommended tolerated dose (MRTD) is the highest dose of an active substance that can be administered chronically to humans without the risk of significant adverse effects. Determining this parameter is helpful, among other things, in establishing safe starting doses for drugs in Phase I clinical trials [50]. The maximum tolerated dose values obtained for compounds **2**–**4**, **6**–**9**, and **11**–**18** were in the range of −0.438 to 0.454, which, according to the pkCSM platform, classifies them as low doses (≤0.477). However, for compounds **1**, **5**, and **10**, values higher than 0.477 were recorded, which allows them to be classified as high.

Acute oral toxicity in rats is the predicted dose of an active substance that after a single oral administration, leads to the death of 50% of the individuals in the tested population [51]. According to the pkCSM prediction model, based on a set of over 10,000 compounds, this parameter is expressed as LD_50_ (mol/kg). Analyzing the results, it was found that all **18** compounds tested had high acute oral toxicity values in rats (ranging from 2.478 to 2.865), with the highest values observed in derivatives with a bromophenyl substituent at the 5-position of the thiazole ring. Chronic oral toxicity in rats refers to repeated and long-term administration of a substance at a dose at which adverse effects are observed [52]. In the pkCSM model, the parameter is predicted as the lowest dose at which harmful effects are observed in animals during long-term exposure (LOAEL). Chronic oral toxicity values for rats obtained in this study ranged from 0.77 to 1.456 log mg/kg/day.

The toxicity parameter for fathead minnows is based on the LC_50_ value, which is the active substance concentration that causes death of 50% of the individuals in the tested population. The calculated logLC_50_ values for pseudothiohydantoin derivatives exceeded the cutoff value of −0.3 (0.5 nM), below which compounds are considered acutely toxic to fathead minnows. Therefore, the compounds in this model are not regarded as toxic. The predicted values of the toxicity parameter for *Tetrahymena pyriformis* ranged from 0.524 to 2.011. The obtained data allow us to conclude that all tested derivatives may be toxic to the single-cell model used, as the values significantly exceed the threshold of −0.5 log µg/L (the negative logarithm of the concentration causing death of 50% of the ciliate population), above which acute toxicity is observed [53]. The Ames test is a commonly used parameter for determining the potential mutagenicity of a chemical compound [54]. All pseudothiohydantoin derivatives tested showed a predicted negative result in the AMES toxicity test, suggesting a lack of mutagenic potential.

## 3. Materials and Methods

The synthesis methods of the studied series of pseudothiohydantoin derivatives and their spectral descriptions have already been described [55,56].

### 3.1. Determiantion of Lipophilicity by RP HPLC

The HPLC studies were performed on the Shimadzu HPLC system (Shimadzu, Kyoto, Japan) equipped with solvent delivery pump LC-20AD, UV-VIS detector model SPD-20A, degasser model DGU-20A5, an column oven model CTO-20A and a column LiChrospher(TM) 100 RP-18 (5 μm), Merck (Hessen, Germany). The mobile phase used consisted of a mixture of methanol and water, the concentration of which was varied in various proportions (water and methanol for HPLC from POCH—Poland). The methanol concentration, expressed as volume fraction (*v*/*v*), was varied gradually from 0.55 to 0.95 in increments of 0.05, and a separate chromatographic analysis was performed for each mobile phase composition. The test compounds were dissolved in methanol at a concentration of 1 mg/mL. The flow rate was 0.7 mL/min, and the injected sample volume was 0.02 mL. All measurements were performed at 25 °C, and detection wavelength of 254 nm was chosen.

The retention factor k was determined according to the formula:

(1) $$k\;=\;\frac{t_{r}}{t_{m}}-1$$

where t_r_ [min]—retention time, and t_m_ [min]—time for dead volume (measured by use of uracil).

In order to determine the lipophilicity parameters, a linear relationship between the logk values and the methanol concentration in the mobile phase was determined according to the following equation:

(2) $$\log k\;=\;\log k_{w}\;-S\varphi ,$$

where log k_w_—value extrapolated to zero methanol concentration, φ—methanol concentration in the mobile phase (volume fraction *v*/*v*), and S—the slope of the regression curve.

The value of parameter φ_0_ was determined according to the formula [57]:

(3) $$\varphi_{0}\;=\;-\frac{logk_{w}}{S}.$$

### 3.2. Determiantion of Lipophilicity by RP TLC

Analyses were performed on HPTLC silica gel 60RP-18 WF254s plates (Merck, Darmstadt, Germany) (10 × 10 cm). A mixture of methanol and water was used as the mobile phase (water and methanol for HPLC from POCH—Poland). The methanol concentration, expressed as a volume fraction (*v*/*v*), was gradually varied from 0.5 to 0.7, at constant intervals of 0.05. Solutions of the tested compounds were prepared in methanol at a 1 mg/mL concentration and then applied to the plates in a volume of 20 µL.

The chromatographic plate was developed in a horizontal DS-chamber (Chromdes, Lublin, Poland), previously saturated with the mobile phase for 20 min. The plates were developed to a distance of 8 cm, then dried at room temperature and analyzed under UV light at 254 nm. All analyses were performed at 25 °C.

For all compounds, the relative lipophilicity values of R_M_ in the five methanol–water phase systems were calculated according to the following equation:

(4) $$R_{M}\;=\log\left[\frac{1-R_{F}}{R_{F}}\right],$$

where R_f_—retention factor.

The lipophilicity parameter R_M0_ was determined based on the linear relationship between the RM values and the methanol concentration in the mobile phase, according to the equation:

(5) $$R_{M}=\;R_{M0}+S\varphi ,$$

where φ—methanol concentration in the mobile phase (volume fraction *v*/*v*), and S—the slope of the regression curve.

The value φ0 was calculated using the following equation [58]:

(6) $$\varphi_{0}\;=\;-\frac{R_{M0}}{S}.$$

### 3.3. In Silico Prediction

The VCCLAB software was used to determine the predicted theoretical partition coefficients for the tested compounds milogP, AlogPs, AClogP, XLOGP2 and XLOGP3 [59].

The publicly available computational platforms SwissADME and pkCSM allowed the determination of all physicochemical parameters and ADMET descriptors [53,60].

Hierarchical cluster analysis (HCA) was performed using only experimental and calculated lipophilicity parameters. The Euclidean distance was used to measure variable variance in the cluster analysis, and the Ward single-linkage method was used to determine the distance between clusters. Statistica 14 software from StatSoft was used for the analysis.

## 4. Conclusions

This paper evaluates 18 new pseudothiohydantoin derivatives regarding lipophilicity, bioavailability parameters, and ADMET determined in silico. The lipophilicity of the studied compounds was experimentally determined using RP-HPLC and RP-TLC methods and then compared with the theoretically determined AlogPs, AClogP, ALOGP, MLOGP, XLOGP2, XLOGP3, and milogP values. For all studied compounds, a linear correlation was obtained between the log k_w_ and R_M0_ values and the concentration of the organic modifier in the mobile phase. The reversed-phase high-performance liquid chromatography method showed stronger correlations with the calculated lipophilicity parameters than the RP-TLC method. Comparative analysis showed that the AClogP and milogP parameters were most similar to the experimentally determined log k_w_ and R_M0_, confirming their usefulness in predicting the lipophilicity of the studied compounds. Bioavailability assessment showed that all derivatives met the Lipinski, Veber, Egan and Ghose rule, indicating potential good oral bioavailability. ADME analysis revealed a favorable pharmacokinetic profile for most of the tested derivatives. Most of the compounds tested showed a favorable safety profile and moderate toxicity. However, it should be emphasized that the results obtained are predictive and require confirmation in future experimental studies.

The results demonstrate that combining chromatographic studies with predictive methods is an effective tool for guiding future research and preliminary evaluation in the drug development process.

## Figures and Tables

**Table 1:** Structures of the studied 2-aminothiazol-4(5*H*)-one derivatives.

No. of Compound	Structural Formula	No. of Compound	Structural Formula	No. of Compound	Structural Formula
**1**	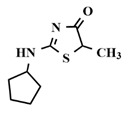	**7**	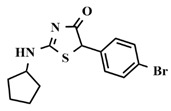	**13**	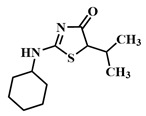
**2**	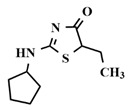	**8**	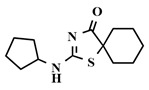	**14**	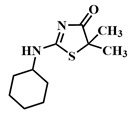
**3**	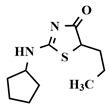	**9**	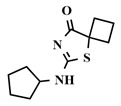	**15**	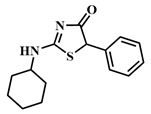
**4**	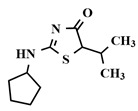	**10**	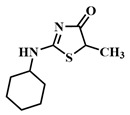	**16**	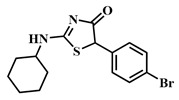
**5**	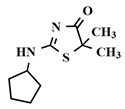	**11**	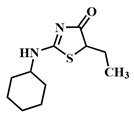	**17**	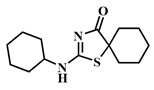
**6**	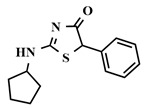	**12**	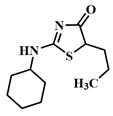	**18**	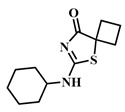

**Table 2:** Lipophilicity parameters determined experimentally using the following methods: RP-HPLC and RP-TLC.

Compound	RP–HPLC				RP–TLC			
	log k_w_	-S	Φ_0_	R	R_M0_	-S	Φ_0_	R
**1**	1.37	2.363	0.580	0.9981	1.93	3.026	0.638	0.9782
**2**	1.83	2.776	0.659	0.9984	2.13	3.305	0.645	0.9872
**3**	2.41	3.291	0.732	0.9984	2.45	3.574	0.685	0.9945
**4**	2.26	3.167	0.714	0.9983	2.26	3.351	0.676	0.9677
**5**	1.80	2.753	0.654	0.9983	2.12	3.267	0.649	0.9503
**6**	2.44	3.441	0.709	0.9975	2.80	3.487	0.803	0.9529
**7**	3.23	4.124	0.783	0.9981	3.26	4.679	0.697	0.9703
**8**	2.89	3.656	0.790	0.9981	2.93	4.121	0.712	0.9947
**9**	2.05	2.899	0.707	0.9976	2.43	3.382	0.717	0.9679
**10**	1.79	2.715	0.659	0.9984	2.04	3.089	0.661	0.9726
**11**	2.27	3.167	0.717	0.9981	2.28	3.388	0.673	0.9629
**12**	2.83	3.664	0.772	0.9981	2.85	4.061	0.701	0.9926
**13**	2.69	3.556	0.756	0.9981	2.81	4.061	0.691	0.9935
**14**	2.22	3.120	0.711	0.9979	2.71	4.109	0.659	0.9892
**15**	3.07	4.091	0.750	0.9974	3.12	4.373	0.714	0.9974
**16**	3.65	4.517	0.808	0.9977	3.70	5.168	0.716	0.9968
**17**	3.33	4.517	0.821	0.9982	3.38	4.657	0.727	0.9993
**18**	2.48	3.289	0.754	0.9978	2.57	3.691	0.697	0.9882

RP-HPLC—reversed phase high performance liquid chromatography; RP-TLC—reversed phase thin layer chromatography; log k_w_—decimal logarithm (log P) of partition coefficient P; S—slope of the regression curve; Φ0—chromatographic lipophilicity parameter; R—correlation coefficient; R_M0_—value extrapolated to zero methanol concentration.

**Table 3:** Lipophilicity parameter values are determined experimentally (log k_w_, R_M0_) and theoretically (milogP, AlogPs, AclogP, XLOGP2, XLOGP3).

Compound	Log k_w_	R_M0_	milogP	AlogPs	AClogP	XLOGP2	XLOGP3
**1**	1.37	1.93	1.48	2.02	1.41	1.20	1.82
**2**	1.83	2.13	1.98	2.53	1.94	1.56	2.34
**3**	2.41	2.45	2.54	2.84	2.47	2.13	2.70
**4**	2.26	2.26	2.23	2.45	2.29	1.86	2.78
**5**	1.80	2.12	1.93	2.39	1.95	1.40	2.01
**6**	2.44	2.80	2.70	3.24	2.70	2.40	3.11
**7**	3.23	3.26	3.51	4.13	3.47	3.20	3.80
**8**	2.89	2.93	3.09	3.08	2.99	2.35	3.02
**9**	2.05	2.43	1.85	2.24	1.87	1.42	2.12
**10**	1.79	2.04	1.99	2.36	1.97	1.77	2.36
**11**	2.27	2.28	2.49	2.87	2.50	2.13	2.89
**12**	2.83	2.85	3.05	3.18	3.04	2.70	3.24
**13**	2.69	2.81	2.73	2.75	2.85	2.43	3.32
**14**	2.22	2.71	2.43	2.82	2.51	1.97	2.55
**15**	3.07	3.12	3.20	3.65	3.26	2.97	3.65
**16**	3.65	3.70	4.01	4.57	4.04	3.77	4.34
**17**	3.33	3.38	3.60	3.46	3.56	2.92	3.56
**18**	2.48	2.57	2.35	2.72	2.43	1.99	2.66

**Table 4:** Spearman and Pearson correlation coefficients.

**Spearman correlation**							
	**logk_w_**	**R_M0_**	**milogP**	**AlogPs**	**AC logP**	**XLOGP2**	**XLOGP3**
**logk_w_**	1.0000	0.9649	0.9567	0.9009	0.9401	0.9499	0.9360
**R_M0_**	0.9649	1.0000	0.9422	0.8968	0.9463	0.9282	0.8989
**milogP**	0.9567	0.9422	1.0000	0.9484	0.9876	0.9768	0.9587
**AlogPs**	0.9009	0.8968	0.9484	1.0000	0.9422	0.9458	0.9133
**AClogP**	0.9401	0.9463	0.9876	0.9422	1.0000	0.9685	0.9505
**XLOGP2**	0.9499	0.9282	0.9768	0.9458	0.9685	1.0000	0.9840
**XLOGP3**	0.9360	0.8989	0.9587	0.9133	0.9505	0.9840	1.0000
**Pearson correlation**							
	**logk_w_**	**R_M0_**	**milogP**	**AlogPs**	**AC logP**	**XLOGP2**	**XLOGP3**
**logk_w_**	1.0000	0.9651	0.9816	0.9139	0.9855	0.9581	0.9558
**R_M0_**	0.9651	1.0000	0.9582	0.9189	0.9634	0.9363	0.9200
**milogP**	0.9816	0.9582	1.0000	0.9483	0.9962	0.9780	0.9639
**AlogPs**	0.9139	0.9189	0.9483	1.0000	0.9450	0.9668	0.9405
**AC logP**	0.9855	0.9634	0.9962	0.9450	1.0000	0.9810	0.9728
**XLOGP2**	0.9581	0.9363	0.9780	0.9668	0.9810	1.0000	0.9876
**XLOGP3**	0.9558	0.9200	0.9639	0.9405	0.9728	0.9876	1.0000

**Table 5:** Physicochemical properties of the obtained series of 2-(cyclopentylamino)thiazol-4(5*H*)-one and 2-(cyclohexylamino)thiazol-4(5*H*)-one derivatives.

Compund	MW [g/mol]	TPSA [Å^2^]	Molar Refractivity	nON	nOHNH	Nrotb
**1**	198.29	66.76	58.75	2	1	2
**2**	212.31	66.76	63.56	2	1	3
**3**	226.34	66.76	68.37	2	1	4
**4**	226.34	66.76	68.37	2	1	3
**5**	212.31	66.76	63.60	2	1	2
**6**	260.35	66.76	78.43	2	1	3
**7**	339.25	66.76	86.13	2	1	3
**8**	252.38	66.76	75.90	2	1	2
**9**	224.32	66.76	66.29	2	1	2
**10**	212.31	66.76	63.56	2	1	2
**11**	226.34	66.76	68.37	2	1	3
**12**	240.36	66.76	73.17	2	1	4
**13**	240.36	66.76	73.17	2	1	3
**14**	226.34	66.76	68.40	2	1	2
**15**	274.38	66.76	83.24	2	1	3
**16**	353.28	66.76	90.94	2	1	3
**17**	266.40	66.76	80.71	2	1	2
**18**	238.35	66.76	71.10	2	1	2

Abbreviations: nOHNH—hydrogen bond donor; nON—hydrogen bond acceptor; TPSA—topological polar surface area; Nrtob—number of rotatable bounds.

**Table 6:** Absorption prediction for pseudothiohydantoin derivatives **1**–**18**.

Compound	Water Solubility [log mol/L]	Caco-2 Permeability [log Papp in 10^−6^ cm/s]	Intestinal Absorption [% Absorbed]	Skin Permeability [log Kp]
**1**	−2.225	1.461	94.414	−3.077
**2**	−2.606	1.461	93.782	−2.93
**3**	−3.007	1.459	92.935	−2.764
**4**	−3.004	1.475	93.294	−2.738
**5**	−2.571	1.463	94.026	−3.011
**6**	−3.783	1.617	92.766	−2.485
**7**	−4.673	1.465	91.317	−2.52
**8**	−4.088	1.47	92.457	−2.503
**9**	−3.293	1.459	93.323	−2.432
**10**	−2.571	1.463	94.026	−3.011
**11**	−2.956	1.463	93.383	−2.872
**12**	−3.357	1.462	92.546	−2.718
**13**	−3.355	1.478	92.905	−2.692
**14**	−2.919	1.466	93.637	−2.949
**15**	−4.119	1.619	92.377	−2.507
**16**	−5.003	1.467	90.929	−2.548
**17**	−4.377	1.472	92.068	−2.527
**18**	−3.595	1.462	92.934	−2.436

**Table 7:** Distribution prediction for pseudothiohydantoin derivatives **1**–**18**.

Compound	VDss [log L/kg]	Fraction Unbound [Fu]	BBB Permeability [log BB]	CNS Permeability [log PS]
**1**	0.123	0.618	−0.208	−3.211
**2**	0.162	0.569	0.137	−3.133
**3**	0.207	0.515	0.126	−3.051
**4**	0.169	0.498	0.153	−2.825
**5**	0.148	0.588	0.163	−3.256
**6**	0.349	0.254	0.229	−2.061
**7**	0.346	0.346	0.199	−2.116
**8**	0.234	0.338	0.221	−2.206
**9**	0.222	0.459	0.222	−2.597
**10**	0.148	0.588	0.163	−3.256
**11**	0.187	0.538	0.15	−3.178
**12**	0.231	0.484	0.139	−3.097
**13**	0.194	0.467	0.166	−2.87
**14**	0.173	0.558	0.176	−3.302
**15**	0.384	0.226	0.242	−2.107
**16**	0.38	0.19	0.212	−2.162
**17**	0.256	0.308	0.234	−2.251
**18**	0.246	0.429	0.184	−2.643

**Table 8:** Metabolism and excretion prediction for pseudothiohydantoin derivatives **1**–**18**.

Compound	Total Clearance [log mL/min/kg]	CYP2D6 Substrate	CYP3A4 Substrate	CYP1A2 Inhibitor	CYP2C19 Inhibitor	CYP2C9 Inhibitor	CYP2D6 Inhibitor	CYP3A4 Inhibitor
**1**	0.168	No	No	No	No	No	No	No
**2**	0.206	No	No	No	No	No	No	No
**3**	0.231	No	No	No	No	Yes	No	No
**4**	0.189	No	No	Yes	No	No	No	No
**5**	0.091	No	No	No	No	No	No	No
**6**	0.042	No	Yes	No	Yes	No	No	No
**7**	−0.111	No	Yes	No	Yes	Yes	No	No
**8**	0.061	No	No	No	No	No	No	No
**9**	−0.025	No	No	No	No	No	No	No
**10**	0.179	No	No	No	No	No	No	No
**11**	0.218	No	No	No	No	No	No	No
**12**	0.243	No	No	No	No	No	No	No
**13**	0.2	No	No	Yes	No	No	No	No
**14**	0.102	No	No	No	No	No	No	No
**15**	0.054	No	Yes	No	Yes	No	No	No
**16**	−0.1	No	Yes	No	Yes	Yes	No	No
**17**	0.073	No	Yes	No	No	No	No	No
**18**	−0.014	No	No	No	No	No	No	No

**Table 9:** Summary of toxicological parameters of the tested pseudothiohydantoin derivatives **1**–**18**.

Compound	Max. Tolerated Dose [log mg/kg/day]	Oral Rat Acute Toxicity [mol/kg]	Oral Rat Chronic Toxicity [log mg/kg/day]	*Tetrahymena pyriformis* Toxicity [log µg/L]	Minnow Toxicity [log mM]	Ames Toxicity
**1**	0.576	2.635	0.896	0.524	2.171	No
**2**	0.425	2.593	0.857	0.808	1.88	No
**3**	0.28	2.555	0.811	1.096	1.626	No
**4**	0.337	2.478	0.875	1.175	1.546	No
**5**	0.515	2.641	0.854	0.718	2.054	No
**6**	−0.374	2.769	1.344	1.874	0.769	No
**7**	−0.287	2.864	0.998	2.209	0.617	No
**8**	−0.326	2.624	1.406	1.052	1.003	No
**9**	−0.025	2.684	1.456	0.742	1.543	No
**10**	0.515	2.641	0.854	0.718	2.054	No
**11**	0.364	2.6	0.816	0.996	1.763	No
**12**	0.219	2.564	0.77	1.275	1.509	No
**13**	0.274	2.486	0.833	1.35	1.429	No
**14**	0.454	2.646	0.813	0.895	1.937	No
**15**	−0.438	2.778	1.303	2.011	0.652	No
**16**	−0.347	2.865	0.918	2.288	0.499	No
**17**	−0.398	2.625	1.365	1.144	0.886	No
**18**	−0.029	2.685	1.414	0.863	1.426	No

## Data Availability

The original contributions presented in this study are included in the article/Supplementary Material. Further inquiries can be directed to the corresponding author.

## Supplementary Materials

The following supporting information can be downloaded at: https://www.mdpi.com/article/10.3390/ijms27146310/s1.
