# Supplementary material for: Integral Lipophilicity Studies with in Silico ADMET Parameter Analysis of Novel Pseudothiohydantoin Derivatives
Source: Int J Mol Sci. 2026 Jul 15;27(14):6310. doi: 10.3390/ijms27146310 (PMC13409817; doi:10.3390/ijms27146310)
Supplement: Supplementary file 1 [file ijms-27-06310-s001.zip › ijms-4400554-supplementary.pdf]

Supplementary Materials

Integral lipophilicity studies with in silico ADMET parameter analysis of novel pseudothiohydantoin derivatives

Szymon Baumgart [1,*], Małgorzata Redka[1], Artur Słomka[2], Renata Studzińska[1]

[1] Department of Organic Chemistry, Faculty of Pharmacy, Collegium Medicum in Bydgoszcz, Nicolaus Copenicus University in Toruń, 2 Jurasza Str., 85-089 Bydgoszcz, Poland; email: sz.baumgart@cm.umk.pl (S.B.); mredka@cm.umk.pl (M.R.); rstud@cm.umk.pl (R.S.);

[2] Department of Hematology and Oncology, National Medical Institute of the Ministry of Interior and Administration, 137 Wołoska Str., 02-507 Warsaw, Poland email: artur.slomka@cm.umk.pl (A.S.);

* Correspondence: sz.baumgart@cm.umk.pl;

Content:

1. Figure S1. Scatter plot showing the correlation of log k_w and R_{M0}.

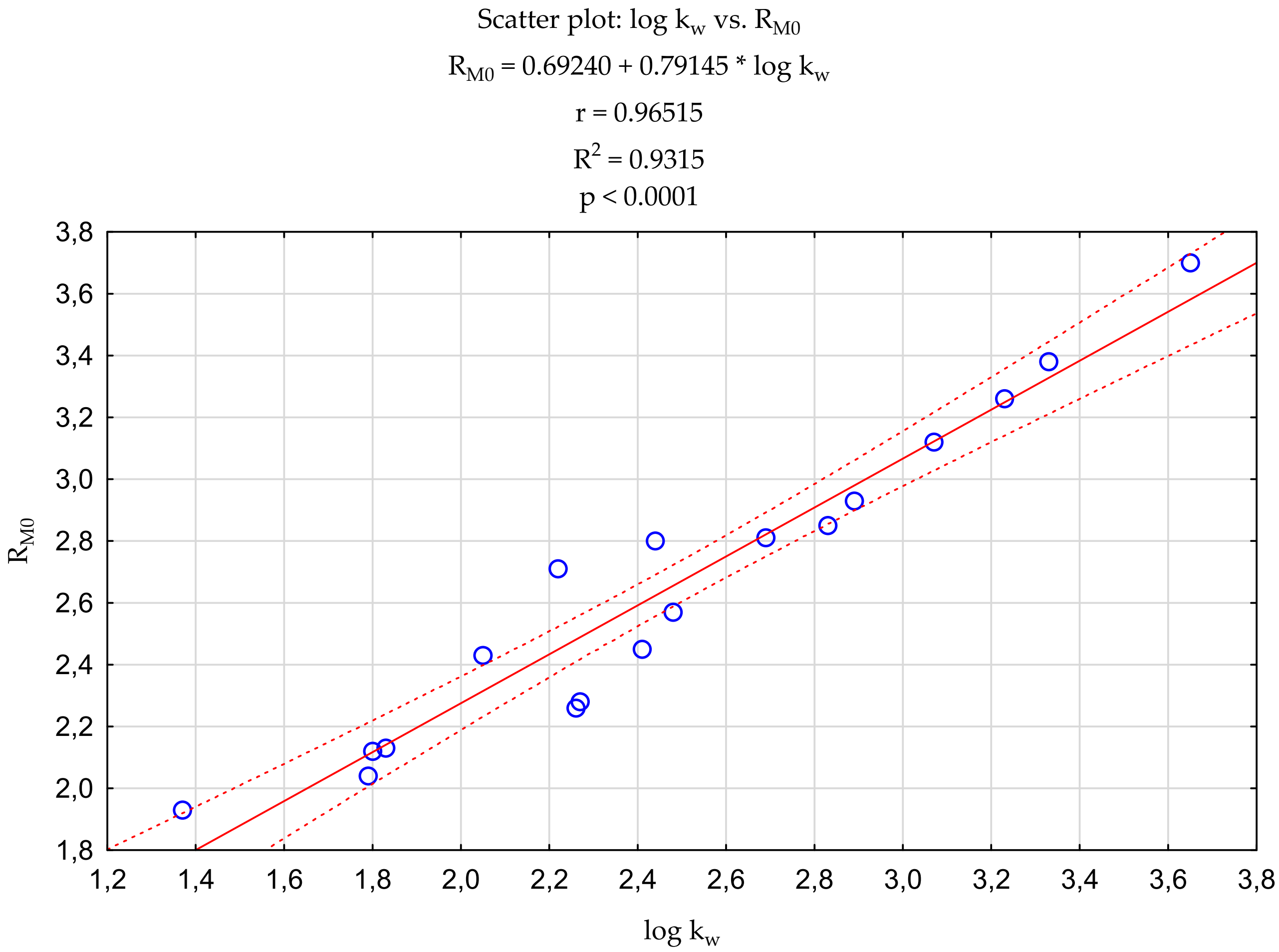

Figure S1. Scatter plot showing the correlation of log k_w and R_{M0}.
